# Criteria for Safe Hospital Discharge in Bronchiolitis: A Systematic Review

**DOI:** 10.1111/1742-6723.70339

**Published:** 2026-09-03

**Authors:** Meredith L. Borland, Kate Loveys, Franz E. Babl, Elizabeth Cotterell, Libby Haskell, Sharon O'Brien, Ed Oakley, Emma J. Tavender, Catherine L. Wilson, Stuart R. Dalziel, the Paediatric Research in Emergency Departments International Collaborative (PREDICT) Network, Jane Alsweiler, David Armstrong, Simon Craig, Nigel Crawford, Dianne Crellin, Sonja Crone, Trevor Duke, Shane George, Christine Jeffries‐Stokes, Nidhi Krishnan, Ken Peacock, Tomas Ratoni, Peter Richmond, Annie Smith, Rebecca Starkie, David Thomas, Alexandra Wallace, Michael Zhang, Marian Chandler, Poh Chua

**Affiliations:** ^1^ Emergency Department Perth Children's Hospital Perth Western Australia Australia; ^2^ Divisions of Paediatrics and Emergency Medicine, School of Medicine University of Western Australia Perth Western Australia Australia; ^3^ Department of Paediatrics: Child and Youth Health, School of Medicine University of Auckland Auckland New Zealand; ^4^ Department of Paediatrics University of Melbourne Parkville Victoria Australia; ^5^ Department of Critical Care University of Melbourne Parkville Victoria Australia; ^6^ Emergency Department Royal Children's Hospital Parkville Victoria Australia; ^7^ Clinical Sciences Murdoch Children's Research Institute Parkville Victoria Australia; ^8^ Armidale Rural Referral Hospital Armidale New South Wales Australia; ^9^ School of Rural Medicine and Tablelands Clinical School University of New England Armidale New South Wales Australia; ^10^ Children's Emergency Department Starship Children's Hospital Auckland New Zealand; ^11^ Division of Emergency Medicine, Anaesthesia and Pain Medicine, Medical School University of Western Australia Perth Western Australia Australia; ^12^ Institute for Paediatric Perioperative Excellence The University of Western Australia Perth Western Australia Australia; ^13^ Australian Catholic University (ACU) Sydney New South Wales Australia; ^14^ Department of Surgery, School of Medicine The University of Auckland Auckland New Zealand

**Keywords:** bronchiolitis, infant, paediatric emergency medicine, paediatrics, patient discharge

## Abstract

Bronchiolitis is the leading cause of hospital presentation and admission for infants in Australasia. We aimed to synthesise current evidence on the effect of discharge criteria for infants (aged < 12 months) who are presenting to or are admitted to hospital with bronchiolitis, to inform a binational guideline recommendation update. Systematic searches were conducted on MEDLINE, EMBASE, PubMed, Cochrane Library and CINAHL (last search 19 February 2025) for non‐randomised studies evaluating hospital discharge criteria in bronchiolitis. The primary outcomes were length of stay (LOS) and readmission rates. The risk of bias (ROBINS‐I) and certainty of the evidence (GRADE) were appraised, and findings were narratively synthesised. GRADE evidence‐to‐decision methodology, expert consensus voting and interest‐holder consultation were used to finalise the recommendation update. Two retrospective observational studies were included (*N* = 2697) (low to very low quality), reporting on unique discharge criteria. In both studies, use of the discharge criteria was associated with a significant reduction in LOS relative to alternative protocols. There was no significant difference in readmission rates observed in either study. There was low to very low certainty evidence across outcomes due to risk of bias, indirectness and imprecision. The review findings informed a recommendation update for safe discharge criteria in the 2025 Australasian Bronchiolitis Guideline update. Updated, prescriptive discharge criteria and flow chart were developed, covering clinical stability, oxygen saturation/support, feeding difficulties, caregiver confidence and education on deterioration, social factors and follow‐up. The revised criteria provide clinicians with increased certainty in decision‐making in bronchiolitis, albeit with further research needed.

## 1 Introduction

Bronchiolitis is the commonest reason for hospital admission, and a frequent cause of emergency department (ED) presentation for infants under 12 months of age in Australia and Aotearoa New Zealand (Australasia). The Australasian Bronchiolitis Guideline (2025) by the Paediatric Research in Emergency Departments International Collaborative (PREDICT) group was undertaken to provide updated evidence‐based clinical guidance for infants presenting to or admitted to hospital with bronchiolitis, targeting clinicians working in EDs, general paediatric wards and intensive care units (ICUs) [1, 2].

At a system level, development of discharge criteria reduces variability in healthcare delivery, supporting consistency in length of stay (LOS) and management approaches across different health services. Prescribed discharge criteria allow clinicians certainty in decision‐making and support planning for individual patients. This is particularly relevant in EDs and in‐patient wards where common conditions such as bronchiolitis are often managed by junior staff. While clinicians value the concept of discharge criteria, particularly for common conditions such as bronchiolitis [3, 4], little work has addressed this question for families in Australasian healthcare settings.

While the first PREDICT Australasian Bronchiolitis Guideline (2016) focused on factors that were a risk for representation rather than discharge criteria, due to insufficient direct evidence to allow for clear recommendations for safe discharge criteria [5], subsequent evidence has been published which may now allow development of discharge criteria recommendations [6, 7]. This paper aims to synthesise current evidence on the effect of discharge criteria for infants who are presenting or admitted to hospital with bronchiolitis.

## 2 Methods

A systematic review was prospectively registered on PROSPERO (CRD42023463917) and reported according to the Preferred Reporting Items for Systematic Reviews and Meta‐Analyses (PRISMA) 2020 statement (Supporting Information Appendix A1) [8]. This is an update of a systematic review that informed the 2016 Australasian Bronchiolitis Guideline discharge criteria recommendation [5, 9].

### 2.1 Search Strategy and Study Selection

A systematic search was performed on electronic databases (Ovid MEDLINE, Ovid EMBASE, CINAHL, PubMed and the Cochrane Library) by a subject librarian (last search 19 February 2025) (Supporting Information Appendix A2), with search limits by English language and publication date (2000 to current). Supplementary manual searches were conducted from reference lists of related articles or guidelines.

The search results were screened against the eligibility criteria, independently in duplicate by 2 of 10 total reviewers using Covidence software (Veritas Health Innovation, Melbourne, Australia). The screening occurred in title and abstract and full‐text stages with training before each stage. Disputes were resolved through consensus with at least one researcher not involved in the initial vote. Subject matter experts checked the final list of included articles to ensure no key studies were missing.

To be included, the study needed to meet the following criteria: (1) Population: infants (aged < 12 months) with a clinical diagnosis of bronchiolitis and/or respiratory syncytial virus (RSV) infection; (2) intervention: any discharge criteria or protocol; (3) comparator: no specific discharge criteria or protocol, care as usual, an alternative protocol; (4) outcomes: reporting on at least one of the following: (i) LOS (primary outcome), (ii) readmission rate (primary outcome); (5) setting: ED, paediatric ward, ICU; (6) study design: non‐randomised studies (randomised studies were thought to be unlikely but could be included if identified); (7) publication characteristics: English language, peer‐reviewed journal articles, published from 2015 to current (noting that articles from the initial review conducted for the 2016 Australasian Bronchiolitis Guideline could include relevant articles from 2000 onwards [5, 9] for the broadened inclusions in 2025). As a result, this review summarises the literature published between 2000 to present. Studies in mixed populations were included where either ≥ 75% of the sample met our population inclusion criteria, or their results were separately reported.

The primary outcomes were defined as: (1) LOS: the duration of hospitalisation from admission to actual or judged ready for discharge; (2) readmission rate: the number of infants readmitted to hospital following a discharge event. These outcomes were prioritised by consensus of the Guideline Advisory Group (GAG) and Guideline Development Committee (GDC) of the Australasian Bronchiolitis Guideline [1].

### 2.2 Data Extraction and Appraisal

The data were extracted into a spreadsheet form by one reviewer (K.L.), and independently reviewed for accuracy by a second reviewer (M.L.B. and E.O.). No automation tools were used, and no additional data or clarification needed to be sought from study authors. Data were extracted on participant characteristics (e.g., demographic and clinical variables), study characteristics (e.g., study design, location, setting, eligibility criteria), intervention and comparator characteristics (e.g., content of discharge criteria), and outcome measures and results. All results that were compatible with each outcome domain were extracted, irrespective of the type of analysis. Readmission rates for any time period were extracted provided they were linked to the initial illness and discharge event by the study authors. LOS data reported in any time unit (hours, days) were extracted.

The risk of bias of the included studies was appraised using the Risk Of Bias In Non‐Randomised Studies—of Interventions (ROBINS‐I) tool (version 1) [10]. One reviewer performed the appraisals (K.L.) using the standard ROBINS‐I form, with independent review by two reviewers (M.L.B. and E.O.). ROBINS‐I provides an overall risk of bias rating of ‘low’, ‘moderate’, ‘serious’, ‘critical’ or ‘no information’ per study based on assessments across seven domains: bias due to confounding, selection of participants, classification of interventions, deviations from intended interventions, missing data, outcome measurement and selection of the reported result. Robvis software was used to visualise the findings [11].

The Grading of Recommendations Assessment, Development and Evaluation (GRADE) method was used to appraise the certainty of the evidence per outcome [12]. The appraisals were made by one reviewer (K.L.) using GRADEpro GDT software [13], and reviewed by a second reviewer (M.L.B., E.O. and S.R.D.). An appraisal of ‘high’, ‘moderate’, ‘low’ and ‘very low’ certainty is made based on whether downgrades are made across domains of risk of bias, inconsistency, indirectness, imprecision and other factors (e.g., publication bias).

### 2.3 Data Synthesis

The findings were synthesised narratively as the included studies were too heterogenous for quantitative synthesis, including in terms of discharge criteria, interventions, populations and setting.

## 3 Results

### 3.1 Study Selection and Characteristics

**FIGURE 1:**
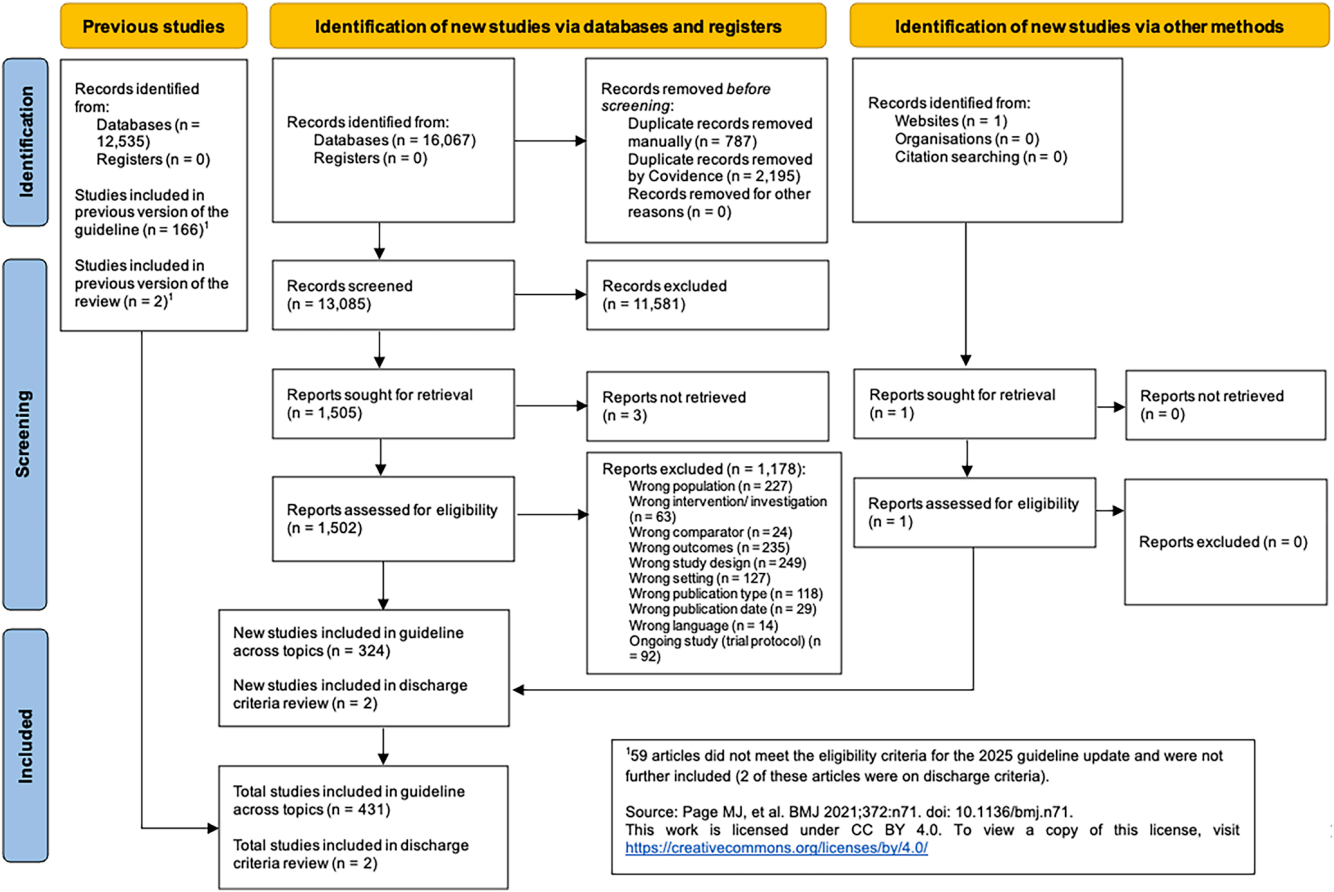
Preferred Reporting Items for Systematic Reviews and Meta‐Analyses (PRISMA) flowchart.

Two of 28,602 records were included in the review (Figure 1) [14, 15]. Related but excluded studies assessed incorrect outcomes [16, 17], such as identifying risk factors for ED readmission as opposed to evaluating discharge criteria. Due to limited evidence, the 2016 Australasian Bronchiolitis Guideline review had included two indirectly relevant studies that did not meet the eligibility criteria of the current review [6, 7]. These studies were not further included.

The included articles for the updated guideline were two retrospective, single‐centre observational studies of bronchiolitis discharge criteria following implementation in hospitals [14, 15]. The studies were conducted in Australia [15] and the USA [14] (*N* = 2697 infants total). Both studies included infants up to 2 years of age, although the majority of infants were aged < 12 months. The US study included predominantly white infants [14], and the Australian study did not report ethnicity data [15]. The detailed study characteristics are presented in Table 1.

**TABLE 1:** Study characteristics.

Study design	Participants	Discharge criteria	Methods	Outcomes and results	Comments
Garcia‐Mauriño et al. [14] *Country*: United States of America *Study type*: Retrospective observational *Location of study*: Nationwide Children's Hospital, Columbus, OH, USA *Study aim*: To assess the impact of a standardised discharge protocol for bronchiolitis on clinical outcomes	*Sample*: *N* = 1813 infants hospitalised with bronchiolitis *n* = 1118 discharged from infectious diseases (ID) unit *n* = 695 discharged from non‐ID units *Characteristics*: Median age: 4 months (IQR 1.8–8.5) (ID unit), 6.5 months (IQR 2.5–12) (non‐ID units) Ethnicity: White 59%, Black non‐Hispanic 23% (ID units); White 55%, Black non‐Hispanic 29% (non‐ID units) *Enrolment setting*: Hospital: ID units, non‐ID units *Key inclusion criteria*: Age: < 2 years of age Discharged with a primary diagnosis of bronchiolitis	*Intervention*: ID unit with a discharge criteria covering: Respiratory status Fever Hydration/intake Social factors Other factors *Comparator*: Non‐ID units that did not use the discharge protocol	Retrospective observational study, electronic health record data	*Length of stay*: Median length of hospital stay in days. Assessed through electronic health record data. ID unit (with protocol) vs. non‐ID units (without protocol): Including PICU stay: 2.0 days (IQR 1.4–3.8) vs. 2.8 days (IQR 1.7–4.9), *p* < 0.001 Adj RR = 1.35, CI = 1.27–1.44, *p* < 0.001 Excluding PICU stay: 1.7 days (IQR 1.1–2.5) vs. 2 days (1.2–3.3), *p* < 0.001 *Readmission rate*: Number of patients readmitted to hospital within 2 weeks of discharge. Assessed through electronic health record data ID unit (with protocol) vs. non‐ID units (without protocol): 33/1118 (2.9%) vs. 18/695 (2.6%), *p* = 0.77 Adj OR = 1.08, CI = 0.57–1.97, *p* = 0.86 *Primary outcomes*: Length of stay Hospital readmission Plus other secondary outcomes reported in the article	Complicated LTRI: PICU stay or parapneumonic effusion or bronchopleural fistula
Gilbert et al. [15] *Country*: Australia *Study type*: Retrospective cohort *Location of study*: The Children's Hospital at Westmead, Sydney, Australia *Study aim*: To assess the safety of revised discharge criteria for infants with bronchiolitis that focused on a shorter duration of monitoring after cessation of oxygen therapy. The secondary aim was to assess the effect of the new discharge criteria on length of stay	*Sample*: *N* = 884 infants hospitalised with bronchiolitis *n* = 462 in 2018 cohort (revised protocol [2019]) *n* = 422 in 2019 cohort (prior protocol [2018]) *Characteristics*: Mean age not reported. 82.9% of the sample were aged ≤ 12 months Ethnicity not reported *Enrolment setting*: Hospital ward *Key inclusion criteria*: Age ≤ 24 months Infants were included who were admitted to hospital with bronchiolitis and who required oxygen therapy from March to October 2018 and 2019 (peak bronchiolitis season) Patients were excluded who were admitted to ICU, < 10 weeks chronological age or > 2 years of age, with a complex and/or chronic medical background (including underlying cardiac, respiratory, neurological, immunological diseases), or who were given complex respiratory support (e.g., CPAP, BiPAP, invasive MV)	*Intervention*: Revised protocol (2019), covering: Respiratory factors (stable during 4 h of monitoring post respiratory support, oxygen saturation, work of breathing/tachypnoea) Feeding and hydration *Comparator*: Prior protocol (2018): Stable during 6 h of monitoring post respiratory support Other aspects of the prior discharge criteria are not reported; unclear whether other components of the discharge criteria were the same as in the revised protocol	Retrospective cohort study, single‐centre. Data were collected from electronic medical records	*Length of stay*: Median length of hospital stay in days (IQR), from admission to discharge Prior protocol vs. revised protocol: Median 2.11 days (IQR 1.54–2.97) vs. 1.83 days (IQR 1.17–2.71), *p* < 0.001 Median difference −6.7 h *Readmission rate*: Number of patients readmitted to hospital within 5 days of discharge, with a diagnosis of bronchiolitis, with respiratory support received in both admissions Prior protocol vs. revised protocol: 3 of 462 (0.6%) vs. 6 of 422 (1.4%), *p* = 0.317 *Primary outcomes*: Safety (comprised of readmission rates, clinical reviews and rapid responses triggered); Plus other secondary outcomes reported in the article	Note, the article reports challenges with protocol adherence and a lack of data on time judged fit for discharge (as opposed to actual discharge, which may have been delayed by factors beyond the protocol). The median time off oxygen to discharge in the 2019 revised protocol year was 423 min (approximately 7 h). Only 71 of 462 (16.7%) patients in the revised protocol cohort were discharged within 4 h of cessation of oxygen therapy. 170 of 462 (36.8%) were discharged within 6 h of cessation of oxygen therapy in the revised protocol cohort

Different discharge criteria were evaluated between the two studies (Table 2). One study looked at the discharge of infants with bronchiolitis from an infectious diseases (IDs) unit using criteria involving respiratory status, fever, hydration/intake, social and other factors [14]. The comparator in this study was discharge from non‐ID units in the same hospital using other unspecified criteria. The second study looked at discharge criteria for infants with bronchiolitis who had received supplementary oxygen during their hospitalisation [15]. The study was primarily interested in the impact of a shorter observation period (4 vs. 6 h) following discontinuation of respiratory support. However, the discharge criteria also included additional variables such as respiratory status and feeding/hydration.

**TABLE 2:** Discharge criteria from the included studies.

Study ID	Discharge criteria
Garcia‐Mauriño et al. [14]	Respiratory status Stable SpO_2_ ≥ 90% in room air for at least 6–12 h for uncomplicated bronchiolitis/LRTI, or at least 24 h for complicated LRTI (PICU stay or parapneumonic effusion or bronchopleural fistula) Improved/no tachypnoea based on WHO definition Fever Afebrile for 24 or 12 h with down trending curve without the use of antipyretics for uncomplicated LRTI, or febrile for at least 24 h without the use of antipyretics for complicated LRTI Hydration/intake Adequate oral intake < 6 months old: ≥ 75% of maintenance fluid requirements 6–12 months old: ≥ 50% of maintenance fluid requirements > 1 year old: ≥ 50% of maintenance fluid requirements or 50% of typical solid intake plus > 25% of maintenance fluid requirements Adequate urine output > 1 mL/kg/h for infants > 0.5 mL/kg/h for children > 1 year old Social Journey home safe criteria satisfied Appropriate care seat, parent training completed for bulb suctioning, assurance of a safe home environment No physician concerns about home environment, inability to comply with therapy, lack of availability to follow up. A follow‐up appointment is made before discharge with the physician's name, phone number and time frame of follow‐up appointment Other factors The patient should have a stable and/or baseline mental status The patient has tolerated their home anti‐infective regimen when necessary
Gilbert et al. [15]	Stable off respiratory support for 4 h^a^ SpO_2_ ≥ 90% Work of breathing or tachypnoea improved to normal or mild severity Adequate feeding and hydration status

Abbreviations: LRTI, lower respiratory tract infection; PICU, paediatric intensive care unit; SpO_2_, peripheral oxygen saturation; WHO, World Health Organisation.

^a^ Continuous pulse oximetry was stopped at 2 h post cessation of respiratory support. Spot saturation checks occurred at 2 and 4 h post cessation of oxygen therapy.

### 3.2 Risk of Bias

**FIGURE 2:**
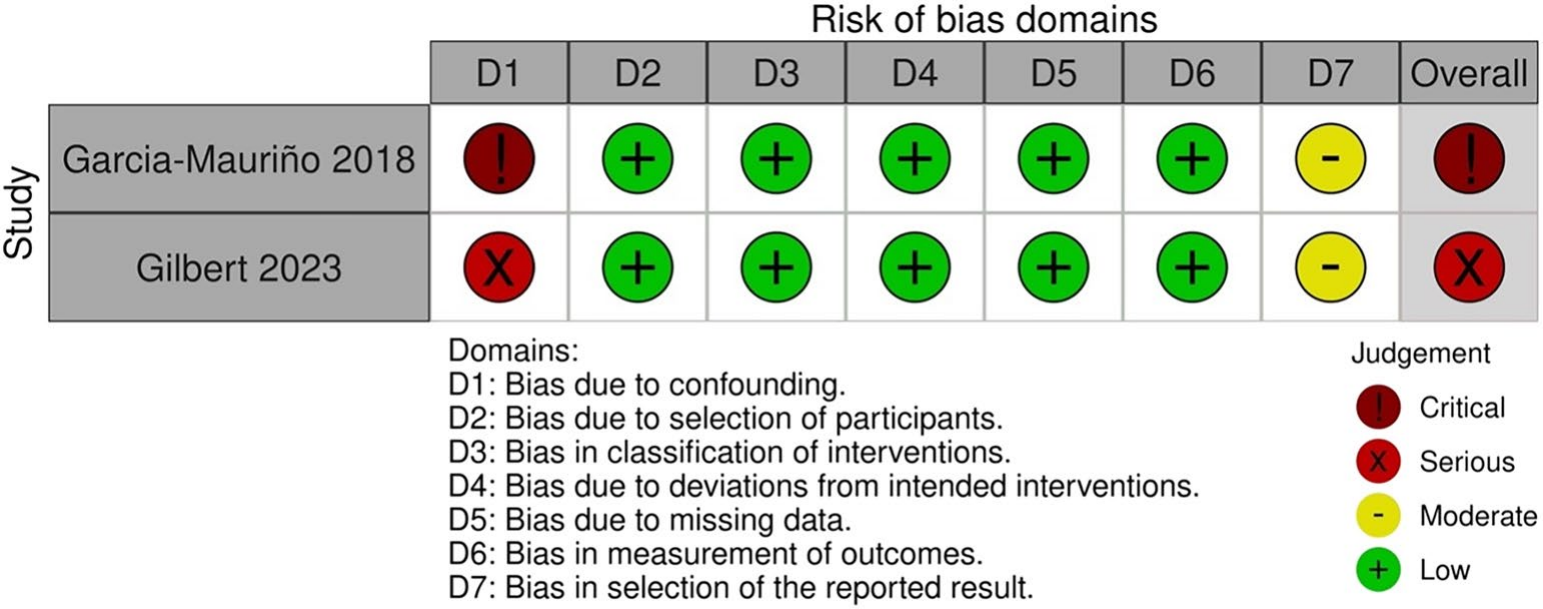
Risk of Bias In Non‐randomised Studies—of Interventions (ROBINS‐I).

The risk of bias appraisals per study are depicted in Figure 2. One study was rated as at critical risk of bias due to critical concerns relating to confounding and moderate concerns about selection of the reported result [14]. The second study was rated at serious risk of bias due to serious concerns about confounding and moderate concerns about selection of the reported result [15].

### 3.3 Length of Stay (LOS)

Both studies found that the evaluated discharge criteria significantly reduced LOS for infants with bronchiolitis (low to very low certainty evidence) [14, 15]. In the US study, infants exposed to the test discharge criteria had a median 0.8 days shorter (*p* < 0.001) hospital stay than infants discharged from other units with alternative criteria (unspecified) [14]. In the Australian study, infants exposed to the test discharge criteria experienced a median 6.7 h shorter (*p* < 0.001) LOS than infants who received the previous criteria [15].

### 3.4 Readmission Rates

There was no significant difference in hospital readmission rates between infants exposed to the test discharge criteria versus the comparator protocol in both studies (very low certainty evidence) [14, 15]. The US study found that 2.9% of infants exposed to the test discharge criteria were readmitted to hospital within 2 weeks of discharge, compared with 2.6% of infants exposed to alternative criteria (*p* = 0.77) [14]. The Australian study reported that 1.4% of infants exposed to the test discharge criteria were readmitted to hospital within 5 days of discharge, with a diagnosis of bronchiolitis requiring further respiratory support [15]. This is compared to 0.6% of infants experiencing this outcome on the prior protocol with a 6‐h observation period (*p* = 0.32).

### 3.5 Certainty of Evidence

Overall, the certainty of evidence for the LOS findings was low to very low due to concerns about risk of bias and indirectness. The evidence for readmission rate was also rated as very low certainty due to concerns about risk of bias, indirectness and imprecision (Supporting Information Appendix A3).

## 4 Discussion

The findings of this review were used as part of the GRADE evidence‐to‐decision framework to update the recommendation on safe discharge criteria within the Australasian Bronchiolitis Guideline (2025 update) [2]. The recommendation was subsequently finalised through consensus voting of the GAG and GDC (*n* = 29 experts), and external interest‐holder consultations [1]. Through the GRADE evidence‐to‐decision framework, these review findings were considered among other factors, such as the feasibility, resource implications, acceptability and health equity implications of our proposed recommendation in Australia and Aotearoa New Zealand hospitals.

The two retrospective observational studies included [14, 15] showed that discharge criteria could reduce LOS without increasing readmission rate, albeit from low to very low‐quality evidence. While the evidence from one study indicated that discharge protocols could significantly reduce LOS, generalisability to the Australasian context is unknown. The second study provided retrospective evidence from an Australian study using discharge criteria post cessation of oxygen therapy with a shortened observation period, demonstrating a significantly shorter LOS. There was no increase in readmissions within 5 days, compared to the standard 6‐h observation protocol.

As there was insufficient evidence to support the recommendation of any specific discharge criteria from these studies, the GAG and GDC additionally considered the discharge criteria that international bronchiolitis guidelines had recommended. A 2021 systematic review of 32 international clinical practice guidelines for bronchiolitis found that only half (*n* = 16) made recommendations on criteria for safe discharge [18]. Some recommendations have subsequently been updated (UK 2021, Canada 2021), and further guidelines have been published (Italy 2022, Egypt 2022). The existing recommendations were generally based on consensus opinion informed by the literature (Table 3).

**TABLE 3:** Discharge criteria recommendations from international bronchiolitis guidelines.

Recommendation	Number of guidelines making recommendation^a^
Improved respiratory effort	9 Guidelines
Improved oxygen saturations (targets > 90% to > 94%)	12 Guidelines
Adequate oral feeding	16 Guidelines
Ability of parent/guardian to cope at home with education	15 Guidelines
Arranged follow up	11 Guidelines

^a^ Derived from Kirolos et al. [18], the updated NICE (UK) 2021 and CPS (Canada) 2021 guidelines, and the ISCD (Italy) 2022 and Egypt 2022 bronchiolitis guidelines.

**FIGURE 3:**
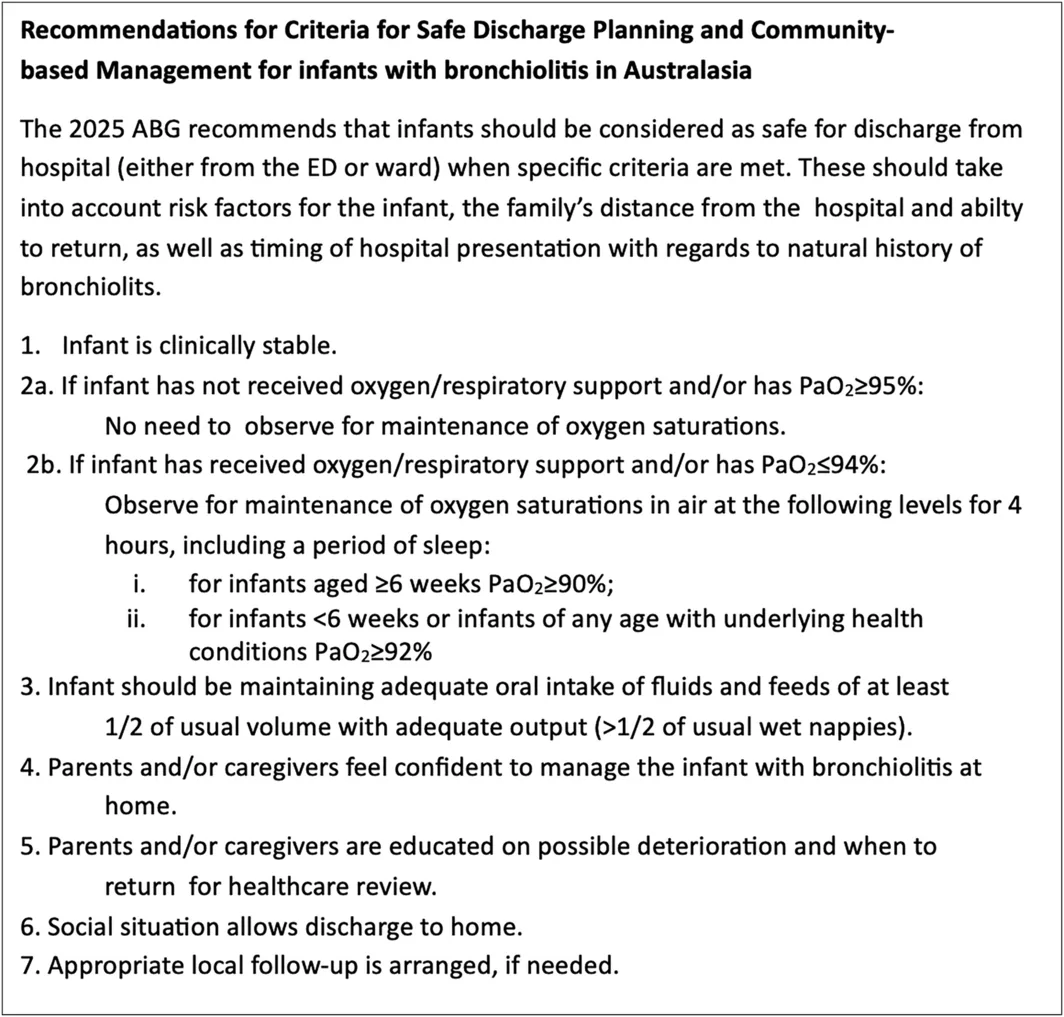
Recommendations for criteria for safe discharge planning and community‐based management for infants with bronchiolitis in Australasia.

Our recommendations were revised considering the eligible evidence and the content of consensus‐based recommendations from existing guidelines. Subsequently, the final recommendation received extensive consultation with interest‐holders, colleges and tertiary paediatric hospitals [1] (Figure 3).

All parents/carers should be provided with key safety information when the infant is discharged, including the expected progression, and when and where to seek further medical care if needed. An understanding of the phase of the illness that the infant is in can help parents and carers anticipate ongoing recovery or potential for further deterioration. A bronchiolitis information sheet (in writing or electronic) should be provided with the capacity to personalise these plans for those patients in circumstances outside of the safe discharge criteria with additional supports in the community.

The GAG and GDC also determined that specifying explicit discharge criteria would enable nurse‐initiated discharge, where appropriate, and thereby reduce LOS. In addition, they determined that further evidence is needed to guide additional criteria for early supported discharge into the community.

### 4.1 Strengths and Limitations

Our review focused on non‐randomised studies of discharge criteria due to the limited evidence identified in the review from the 2016 guideline [5]. There were no RCTs available for review in this area; however, this was expected, and it may not be feasible or ethical to design an RCT to develop higher quality evidence for these criteria. In addition, the limited number of included studies limits the applicability to the broad range of sociodemographic diversity in the Australasian communities to understand how these criteria would fare for all families.

There was insufficient evidence for any specific criteria to develop different discharge recommendations for specific subgroups; for example, those infants discharged from an in‐patient unit who may or may not have received respiratory support.

### 4.2 Future Research

Future research on safe discharge criteria in bronchiolitis should involve co‐design with parents and families, and high‐quality evaluations. Research is needed in diverse subgroups (e.g., varying infant age, socioeconomic status, backgrounds) in the Australasian setting.

## 5 Conclusion

This systematic review informed a recommendation update for evidence‐based, safe discharge from hospital for infants with bronchiolitis in Australasia. The updated criteria cover a range of important clinical and social factors and are prescriptive in design to provide clinicians with increased certainty in decision‐making. However, further research is needed to strengthen the limited evidence base, including more local evaluations with families of more diverse sociodemographic backgrounds.

## Funding

This research was conducted as part of the PREDICT Australasian Bronchiolitis Guideline 2025 update, which was developed with funding support from the National Health and Medical Research Council (NHMRC) Centre of Research Excellence grant for Paediatric Emergency Medicine (GNT1171228), Canberra, Australia, administered by Murdoch Children's Research Institute in Melbourne, VIC, Australia. Stuart R. Dalziel, Kate Loveys and Libby Haskell's time has been part‐funded by Cure Kids New Zealand. Franz E. Babl's time has been part‐funded by a grant from the Royal Children's Hospital Foundation, Parkville, Australia, and an NHMRC Leadership grant, Canberra, Australia. The views of the funders have not influenced the development or content of the research.

## Conflicts of Interest

Meredith L. Borland and Stuart R. Dalziel have been provided with investigational product from Amphastar Pharmaceuticals Company (epinephrine inhalers), USA and Trudell Medical (spacers), Canada, for a study of dexamethasone and epinephrine in bronchiolitis. The other authors declare no conflicts of interest.

## Supporting information


**Appendix A1.** PRISMA 2020 checklist.
**Appendix A2**. Systematic search strategies.
**Appendix A3**. GRADE certainty of evidence tables.

## Data Availability

The data that support the findings of this study are available from the corresponding author upon reasonable request.
