# Supplementary material for: Criteria for Safe Hospital Discharge in Bronchiolitis: A Systematic Review
Source: Emerg Med Australas. 2026 Sep 3;38(5):e70339. doi: 10.1111/1742-6723.70339 (PMC13539362; doi:10.1111/1742-6723.70339)
Supplement: Supplementary file 1 [file EMM-38-0-s001.docx]

Borland ML, Loveys K, Babl FE, Cotterell E, Haskell L, O’Brien S, Oakley E, Tavender EJ, Wilson CL, Dalziel SR, on behalf of the Paediatric Research in Emergency Departments International Collaborative (PREDICT) Network. Criteria for safe hospital discharge in bronchiolitis: a systematic review. Emergency Medicine Australasia. 2026.

# Supplementary material

Supplementary Appendix [A1. PRISMA 2020 Checklist	2](#_Toc225328837)

Supplementary [Appendix A2. Systematic search strategies	8](#_Toc225328838)

Supplementary [Appendix A3. GRADE certainty of evidence tables	29](#_Toc225328839)

## **Supplementary Appendix A1. PRISMA 2020 Checklist**

# PRISMA 2020 Main Checklist

| **Topic** | **No.** | **Item** | **Location where item is reported** |
| --- | --- | --- | --- |
| **TITLE** |  |  |  |
| **Title** | 1 | Identify the report as a systematic review. | Title page |
| **ABSTRACT** |  |  |  |
| **Abstract** | 2 | See the PRISMA 2020 for Abstracts checklist |  |
| **INTRODUCTION** |  |  |  |
| **Rationale** | 3 | Describe the rationale for the review in the context of existing knowledge. | Introduction > paragraphs 2-3 |
| **Objectives** | 4 | Provide an explicit statement of the objective(s) or question(s) the review addresses. | Introduction > paragraph 3 |
| **METHODS** |  |  |  |
| **Eligibility criteria** | 5 | Specify the inclusion and exclusion criteria for the review and how studies were grouped for the syntheses. | Methods > search strategy and study selection > paragraph 3 |
| **Information sources** | 6 | Specify all databases, registers, websites, organisations, reference lists and other sources searched or consulted to identify studies. Specify the date when each source was last searched or consulted. | Methods > search strategy and search strategy > paragraph 1 |
| **Search strategy** | 7 | Present the full search strategies for all databases, registers and websites, including any filters and limits used. | Methods > appendix 2 |
| **Selection process** | 8 | Specify the methods used to decide whether a study met the inclusion criteria of the review, including how many reviewers screened each record and each report retrieved, whether they worked independently, and if applicable, details of automation tools used in the process. | Methods > search strategy and study selection > paragraph 2 |
| **Data collection process** | 9 | Specify the methods used to collect data from reports, including how many reviewers collected data from each report, whether they worked independently, any processes for obtaining or confirming data from study investigators, and if applicable, details of automation tools used in the process. | Methods > data extraction and appraisal > paragraph 1 |
| **Data items** | 10a | List and define all outcomes for which data were sought. Specify whether all results that were compatible with each outcome domain in each study were sought (e.g. for all measures, time points, analyses), and if not, the methods used to decide which results to collect. | Methods > search strategy and study selection > paragraph 3 |
|  | 10b | List and define all other variables for which data were sought (e.g. participant and intervention characteristics, funding sources). Describe any assumptions made about any missing or unclear information. | Methods > data extraction and appraisal > paragraph 1 |
| **Study risk of bias assessment** | 11 | Specify the methods used to assess risk of bias in the included studies, including details of the tool(s) used, how many reviewers assessed each study and whether they worked independently, and if applicable, details of automation tools used in the process. | Methods > data extraction and appraisal > paragraph 1 |
| **Effect measures** | 12 | Specify for each outcome the effect measure(s) (e.g. risk ratio, mean difference) used in the synthesis or presentation of results. | Methods > data synthesis > paragraph 1 |
| **Synthesis methods** | 13a | Describe the processes used to decide which studies were eligible for each synthesis (e.g. tabulating the study intervention characteristics and comparing against the planned groups for each synthesis (item 5)). | Methods > data synthesis > paragraph 1 |
|  | 13b | Describe any methods required to prepare the data for presentation or synthesis, such as handling of missing summary statistics, or data conversions. | Methods > data synthesis > paragraph 1 |
|  | 13c | Describe any methods used to tabulate or visually display results of individual studies and syntheses. | Methods > data synthesis > paragraph 1 |
|  | 13d | Describe any methods used to synthesize results and provide a rationale for the choice(s). If meta-analysis was performed, describe the model(s), method(s) to identify the presence and extent of statistical heterogeneity, and software package(s) used. | Methods > data synthesis > paragraph 1 |
|  | 13e | Describe any methods used to explore possible causes of heterogeneity among study results (e.g. subgroup analysis, meta-regression). | NA |
|  | 13f | Describe any sensitivity analyses conducted to assess robustness of the synthesized results. | NA |
| **Reporting bias assessment** | 14 | Describe any methods used to assess risk of bias due to missing results in a synthesis (arising from reporting biases). | Methods > data extraction and appraisal > paragraph 2 |
| **Certainty assessment** | 15 | Describe any methods used to assess certainty (or confidence) in the body of evidence for an outcome. | Methods > data extraction and appraisal > paragraph 3 |
| **RESULTS** |  |  |  |
| **Study selection** | 16a | Describe the results of the search and selection process, from the number of records identified in the search to the number of studies included in the review, ideally using a flow diagram. | Results > Study selection and characteristics > paragraph 1, figure 1 |
|  | 16b | Cite studies that might appear to meet the inclusion criteria, but which were excluded, and explain why they were excluded. | Results > Study selection and characteristics > paragraph 1 |
| **Study characteristics** | 17 | Cite each included study and present its characteristics. | Table 1 |
| **Risk of bias in studies** | 18 | Present assessments of risk of bias for each included study. | Results > risk of bias > paragraph 1; figure 2 |
| **Results of individual studies** | 19 | For all outcomes, present, for each study: (a) summary statistics for each group (where appropriate) and (b) an effect estimate and its precision (e.g. confidence/credible interval), ideally using structured tables or plots. | Table 1 |
| **Results of syntheses** | 20a | For each synthesis, briefly summarise the characteristics and risk of bias among contributing studies. | Results > Length of stay subsection, Readmission rates subsection |
|  | 20b | Present results of all statistical syntheses conducted. If meta-analysis was done, present for each the summary estimate and its precision (e.g. confidence/credible interval) and measures of statistical heterogeneity. If comparing groups, describe the direction of the effect. | NA |
|  | 20c | Present results of all investigations of possible causes of heterogeneity among study results. | NA |
|  | 20d | Present results of all sensitivity analyses conducted to assess the robustness of the synthesized results. | NA |
| **Reporting biases** | 21 | Present assessments of risk of bias due to missing results (arising from reporting biases) for each synthesis assessed. | NA |
| **Certainty of evidence** | 22 | Present assessments of certainty (or confidence) in the body of evidence for each outcome assessed. | Results > Certainty of evidence section, Appendix 3 |
| **DISCUSSION** |  |  |  |
| **Discussion** | 23a | Provide a general interpretation of the results in the context of other evidence. | Discussion > Paragraph 3 |
|  | 23b | Discuss any limitations of the evidence included in the review. | Discussion > Strengths and Limitations subsection |
|  | 23c | Discuss any limitations of the review processes used. | Discussion > Strengths and Limitations subsection |
|  | 23d | Discuss implications of the results for practice, policy, and future research. | Discussion > paragraphs 1, 4-6; Future research subsection; Conclusion |
| **OTHER INFORMATION** |  |  |  |
| **Registration and protocol** | 24a | Provide registration information for the review, including register name and registration number, or state that the review was not registered. | Methods > paragraph 1 |
|  | 24b | Indicate where the review protocol can be accessed, or state that a protocol was not prepared. | Methods > paragraph 1 |
|  | 24c | Describe and explain any amendments to information provided at registration or in the protocol. | Methods > paragraph 1 |
| **Support** | 25 | Describe sources of financial or non-financial support for the review, and the role of the funders or sponsors in the review. | Funding statement |
| **Competing interests** | 26 | Declare any competing interests of review authors. | Conflicts of interest statement |
| **Availability of data, code and other materials** | 27 | Report which of the following are publicly available and where they can be found: template data collection forms; data extracted from included studies; data used for all analyses; analytic code; any other materials used in the review. | Data availability statement |

# PRIMSA Abstract Checklist

| **Topic** | **No.** | **Item** | **Reported?** |
| --- | --- | --- | --- |
| **TITLE** |  |  |  |
| **Title** | 1 | Identify the report as a systematic review. | Yes |
| **BACKGROUND** |  |  |  |
| **Objectives** | 2 | Provide an explicit statement of the main objective(s) or question(s) the review addresses. | Yes |
| **METHODS** |  |  |  |
| **Eligibility criteria** | 3 | Specify the inclusion and exclusion criteria for the review. | Yes |
| **Information sources** | 4 | Specify the information sources (e.g. databases, registers) used to identify studies and the date when each was last searched. | Yes |
| **Risk of bias** | 5 | Specify the methods used to assess risk of bias in the included studies. | Yes |
| **Synthesis of results** | 6 | Specify the methods used to present and synthesize results. | Yes |
| **RESULTS** |  |  |  |
| **Included studies** | 7 | Give the total number of included studies and participants and summarise relevant characteristics of studies. | Yes |
| **Synthesis of results** | 8 | Present results for main outcomes, preferably indicating the number of included studies and participants for each. If meta-analysis was done, report the summary estimate and confidence/credible interval. If comparing groups, indicate the direction of the effect (i.e. which group is favoured). | Yes |
| **DISCUSSION** |  |  |  |
| **Limitations of evidence** | 9 | Provide a brief summary of the limitations of the evidence included in the review (e.g. study risk of bias, inconsistency and imprecision). | Yes |
| **Interpretation** | 10 | Provide a general interpretation of the results and important implications. | Yes |
| **OTHER** |  |  |  |
| **Funding** | 11 | Specify the primary source of funding for the review. | Yes |
| **Registration** | 12 | Provide the register name and registration number. | Yes |

*From:* Page MJ, McKenzie JE, Bossuyt PM, Boutron I, Hoffmann TC, Mulrow CD, et al. The PRISMA 2020 statement: an updated guideline for reporting systematic reviews. MetaArXiv. 2020, September 14. DOI: 10.31222/osf.io/v7gm2. For more information, visit: [www.prisma-statement.org](http://www.prisma-statement.org).

## **Supplementary Appendix A2. Systematic search strategies**

**Ovid MEDLINE(R) ALL <1946 to June 19, 2023>**

Original search date: 21/06/23

1	bronchiolitis/ or bronchiolitis, viral/ or respiratory syncytial viruses/ or respiratory syncytial virus, human/ or Respiratory Syncytial Virus Infections/ or (bronchiolit* or wheez* or (Respiratory adj1 Syncytial adj1 Virus*)).af. or rsv.tw.	48765

2	limit 1 to (case reports or comment or editorial or letter)	6402

3	limit 1 to (clinical trial, all or clinical trial, phase i or clinical trial, phase ii or clinical trial, phase iii or clinical trial, phase iv or clinical trial or controlled clinical trial or guideline or meta analysis or practice guideline or randomized controlled trial or "review" or systematic reviews)	8638

4	1 and exp Evidence-Based Medicine/	135

5	(1 not 2) or 3 or 4	42783

6	*bronchiolitis, viral/bl, ci, cl, co, dg, di, dt, ep, et, ge, hi, im, mi, mo, pa, pp, pc, th, ur, vi	1012

7	*bronchiolitis/bl, ci, cl, co, dg, di, dt, ep, et, ge, hi, im, mi, mo, pa, pp, pc, th, ur, vi	2446

8	6 or 7	3450

9	limit 8 to (case reports or comment or editorial or letter)	805

10	limit 8 to (clinical trial, all or clinical trial, phase i or clinical trial, phase ii or clinical trial, phase iii or clinical trial, phase iv or clinical trial or controlled clinical trial or guideline or meta analysis or practice guideline or randomized controlled trial or "review" or systematic reviews)	877

11	8 and exp Evidence-Based Medicine/	64

12	(8 not 9) or 10 or 11	2702

13	Natural History/ or exp Epidemiology/	29277

14	exp "reproducibility of results"/ or (scoring adj1 system*).tw.	503239

15	"severity of illness index"/ or (disease adj1 severity).tw.	311661

16	diagnosis, differential/	467890

17	physical examination/ or exp auscultation/ or blood pressure determination/ or exp palpation/ or percussion/ or pulse/ or exp vital signs/	517012

18	exp *Respiratory Tract Infections/	529915

19	risk factors/	955289

20	"length of stay"/ or patient admission/ or patient discharge/ or (criteria adj4 discharge).tw.	156283

21	exp intensive care units, pediatric/ or respiratory care units/ or (nicu or icu or picu or intensive-care).tw.	231239

22	morbidity/ or prevalence/ or exp mortality/	781380

23	exp Diagnostic Imaging/ or radiography, thoracic/ or ((chest adj1 x-ray*) or (chest adj1 xray*)).tw.	2929342

24	exp Hematologic Tests/ or ((blood adj1 test*) or (blood adj1 exam*) or (virologic* adj1 investigation*)).tw.	297077

25	Urinalysis/	9268

26	Nasal Lavage Fluid/ or ((nose or nasal) adj1 (mucus or mucosa)).tw.	10677

27	Emergency Service, Hospital/ or (emergency adj1 department*).tw.	157373

28	asthma/ or hypersensitivity, immediate/ or (atopy or atopic).tw.	186484

29	(salbutamol or ventolin or levalbuterol or adrenalin* or epinephrin* or (beta* adj2 adrenergic*) or (beta* adj2 agonist*) or ics or (inhaled adj1 corticosteroid*) or montelukast).tw.	133389

30	exp Albuterol/ad, tu	7239

31	exp Epinephrine/ad, tu	10031

32	exp Bronchodilator Agents/ad, tu or bronchodilat*.tw.	63186

33	exp steroids/ad, tu	245236

34	exp Cholinergic Antagonists/ or receptors, adrenergic, beta-2/ or (cholinergic adj1 receptor adj1 block* adj1 agent*).tw.	91309

35	exp Anti-Inflammatory Agents/	568228

36	exp Adrenal Cortex Hormones/ or (corticosteroid* or (cortico adj1 steroid*) or glucocorticoid* or gluco corticoid*).tw.	513603

37	Leukotriene Antagonists/ or (Leukotriene adj1 receptor adj1 block* adj1 agent*).tw.	3242

38	Saline Solution, Hypertonic/ad, tu or (hypertonic and (saline adj1 solution)).tw.	3206

39	exp Aerosols/ and exp Sodium Chloride/	410

40	exp "nebulizers and vaporizers"/ and exp sodium chloride/	116

41	((aerosoli#ed adj1 saline) or (nebuli#ed adj1 saline)).tw.	203

42	exp Oxygen Inhalation Therapy/ or *Oxygen/ad, st [Administration & Dosage, Standards]	30943

43	(exp Oximetry/ or oximet*.tw.) and (exp "reproducibility of results"/ or (reliability or function or (technical adj1 specification*) or (percutaneous adj1 measurement*)).tw. or exp blood gas analysis/ or ((pulse adj1 oximet*) or (supplementa* adj1 oxygen) or (oxygen adj1 saturation) or (oxygen adj1 therap*) or (oxygen adj1 treatment*)).tw.)	22242

44	continuous positive airway pressure/ or positive pressure respiration/ or (bubble adj1 CPAP).tw.	27198

45	exp Physical Therapy Modalities/	178123

46	Physical Therapy Specialty/ or physical therapists/	5873

47	(physiotherap* or (physical adj therap*)).tw.	59298

48	((nasal* or nose or naso) adj3 suction*).tw.	122

49	suction/ or (deep adj1 suction*).tw.	13138

50	saline.tw. and Administration, Intranasal/	756

51	((saline adj1 drop*) or (nasal adj1 saline)).tw.	483

52	exp Fluid Therapy/ or Rehydration Solutions/	22592

53	enteral nutrition/ or exp parenteral nutrition/ or intubation, gastrointestinal/	50460

54	(((non adj1 oral) or oral) and (feed* or hydration or fluid* or solution* or therap*)).tw.	211378

55	exp bacterial infections/	961373

56	exp otitis media/	25757

57	exp Meningitis/	58718

58	exp *anti-bacterial agents/tu or (antibiotic* or 3z or aruzilina or atizor or azadose or azasite or azatril or azenil or azibiot or azibiot-neo or azimin or azithral or azithromycin or azitrocin or azitromax or azitromicin or azitromicina or aziwok or azomyne or aztrin or azydrop or azyter or azythromycin or bazyt or cp-62933 or cp-62993 or cp62933 or cp62993 or forcin or inedol or infectoazit or isv-401 or isv401 or kromicin or macrozit or mezatrin or octavax or ordipha or ribotrex or sumamed or sunamed or tobyl or tromix or trozocina or ultreon or vinzam or xithrone or xz-450 or xz450 or zaret or zarom or zetamax or zeto or zibramax or zifin or zimericina or zistic or zithromax or zithrox or zitinn or zitrim or zitrobifan or zitrocin or zitromax or zmax).tw.	490886

59	exp Sepsis/	141157

60	exp Urinary Tract Infections/	50669

61	Tracheitis/	1560

62	(serious adj1 bacterial adj1 infection*).tw.	1294

63	infection control/ or exp primary prevention/ or patient isolation/	213304

64	COVID-19/ or SARS-CoV-2/	234537

65	(2019-novel or 2019nCoV or 2019-nCoV or COVID-19 or COVID19 or COVID-2019 or COVID2019 or CONVID-19 or CONVID19 or CORVID-19 or CORVID19 or CoV2 or CoV-2 or HCoV* or Ncov* or Ncorona* or Ncorono* or NcovChina* or NcovChinese* or NcovHubei* or NcovWuhan* or SARS2 or SARS-2 or SARScoronavirus2 or SARScoronavirus-2 or SARScoronovirus2 or SARScoronovirus-2 or SARSCov19 or SARSCov-19 or SARS-CoV-2 or SARSCoV-2 or SARSCoV2 or WN-CoV or WNCoV or wuhan-virus).tw.	341860

66	((pneumonia* or outbreak* or respiratory-illness* or respiratory-disease* or respiratory-symptom* or seafood-market* or food-market* or wildlife) and (Wuhan or China or Chinese or Hubei or Huanan)).tw.	21170

67	((new or novel or nouveau or risk factors or "2019" or Wuhan or Hubei or Huanan or China or Chinese) adj3 (coronavirus* or corona virus* or betacoronavirus* or CoV or HCoV)).tw.	75998

68	POST-ACUTE COVID-19 SYNDROME/ or (longCOVID* or postCOVID* or postcoronavirus* or postSARS*).tw.	2221

69	(coronavirus/ or betacoronavirus/ or coronavirus infections/) and (disease outbreaks/ or epidemics/ or pandemics/)	40238

70	((coronavirus* or corona-virus* or betacoronavirus*) adj3 (pandemic* or epidemic* or outbreak* or crisis)).tw.	15507

71	exp Antibodies, Monoclonal/ or (monoclonal-antibod* or clonal-antibod* or hybridoma-antibod* or nirsevimab or medi-8897 or medi8897 or sp-0232 or sp-232 or sp0232 or sp232 or Motavizumab or medi-524 or medi524 or numax or Palivizumab or abbosynagis or medi-493 or medi493 or synagis or synagys).tw.	362383

72	or/13-71	9536442

73	5 and 72	28221

74	exp pregnant women/ or exp pregnancy/ or prenatal care/ or (parturition or ante-natal or antenatal* or pre-natal* or prenatal* or pregnan*).tw.	1176170

75	exp immunization/ or (immunis* or immuniz* or vaccin*).tw.	554939

76	(Respiratory-syncytial-virus-vaccine* or RSV-vaccine* or Arexvy).tw.	910

77	(74 and 75 and 5) or (74 and 76)	271

78	(75 and 5) or 76	4709

79	12 or 73 or 78	29996

80	(newborn* or new-born* or baby or babies or neonat* or neo-nat* or infan* or toddler* or aged-1 or aged-one or one-year-old or 1-year-old or under-two or under-2 or younger-than-two or younger-than-2 or below-two or below-2 or under-24-months or younger-than-24-months or below-24-months or aged-1-to-23-months or aged-one-to-twenty-three-months).tw,kf,hw.	1692458

81	exp Bronchiolitis Obliterans/ or (bronchiolitis adj1 obliterans).af.	5735

82	(79 and 80) not 81	13211

83	77 not 81	271

84	82 or 83	13251

85	limit 84 to (english language and yr="2000 -Current")	9850

**Embase <1974 to 2023 June 19>**

Original search date: 21/06/23

1	bronchiolitis/ or viral bronchiolitis/ or exp human respiratory syncytial virus/ or respiratory syncytial virus infection/ or (bronchiolit* or wheez* or (Respiratory adj1 Syncytial adj1 Virus*)).af. or rsv.tw.	93187

2	limit 1 to (editorial or letter or note)	7059

3	1 and (exp controlled clinical trial/ or exp practice guideline/ or meta analysis/ or "review"/ or "systematic review"/)	16414

4	1 and exp evidence based medicine/	6467

5	(1 not 2) or 3 or 4	86742

6	*bronchiolitis/co, di, dm, dr, dt, ep, et, pc, rt, th	3796

7	*viral bronchiolitis/co, di, dm, dr, dt, ep, et, pc, th	548

8	6 or 7	4336

9	limit 8 to (editorial or letter or note)	571

10	8 and (exp controlled clinical trial/ or exp practice guideline/ or meta analysis/ or "review"/ or "systematic review"/)	1137

11	8 and exp evidence based medicine/	555

12	(8 not 9) or 10 or 11	3866

13	History/ or exp Epidemiology/	4735239

14	reproducibility/ or exp validity/ or scoring system/ or exp reliability/	847991

15	exp disease severity/ or (severity adj2 illness).tw.	2249656

16	differential diagnosis/ or physical examination/ or palpation/ or percussion/ or blood pressure measurement/ or blood pressure monitoring/ or pulse rate/ or vital sign/ or ascultation.tw.	813575

17	exp *respiratory tract infection/	235051

18	risk factor/	1336657

19	"length of stay"/ or hospital admission/ or hospital discharge/ or (criteria adj4 discharge).tw.	638513

20	intensive care unit/ or medical intensive care unit/ or neonatal intensive care unit/ or pediatric intensive care unit/ or (nicu or icu or picu or intensive-care).tw.	452434

21	morbidity/ or prevalence/ or mortality/ or childhood mortality/ or infant mortality/	2024864

22	exp diagnostic imaging/ or exp thorax radiography/ or ((chest adj1 x-ray*) or (chest adj1 xray*)).tw.	485079

23	exp blood examination/ or ((blood adj1 test*) or (haem* adj1 exam*) or (haem* adj1 test*) or (virologic* adj1 investigation*)).tw.	370894

24	exp urinalysis/	127990

25	nose mucus/ or nose mucosa/ or (nasal adj1 lavage adj1 fluid*).tw.	18815

26	(emergency adj1 department*).tw.	193543

27	exp asthma/ or atopy/	315735

28	(salbutamol or ventolin or levalbuterol or adrenalin* or epinephrin* or (beta* adj2 adrenergic*) or (beta* adj2 agonist*) or ics or (inhaled adj1 corticosteroid*) or montelukast).tw.	178202

29	salbutamol/ad, do, dt	18740

30	epinephrine/ad, do, dt	5593

31	exp bronchodilating agent/ad, do, dt or bronchodilat*.tw.	99057

32	exp steroid/ad, do, dt	649505

33	exp cholinergic receptor blocking agent/ or (cholinergic adj1 antagonist*).tw.	318374

34	exp antiinflammatory agent/	2480733

35	exp corticosteroid/ or (corticosteroid* or (cortico adj1 steroid*) or glucocorticoid* or gluco corticoid*).tw.	1152309

36	exp leukotriene receptor blocking agent/ or (leukotriene adj1 antagonist*).tw.	23668

37	(sodium chloride/ad, do, dt and (aerosol/ or hypertonic solution/ or exp nebulizer/ or vaporizer/)) or (hypertonic and (saline adj1 solution)).tw.	1308

38	((aerosoli#ed adj1 saline) or (nebuli#ed adj1 saline)).tw.	284

39	exp oximetry/ or exp oxygen therapy/ or oxygen/ad, do	124833

40	(exp oximetry/ or oximet*.tw.) and (reproducibility/ or (reliability or function or (technical adj1 specification*) or (percutaneous adj1 measurement*)).tw. or exp blood gas analysis/ or ((pulse adj1 oximet*) or (supplementa* adj1 oxygen) or (oxygen adj1 saturation) or (oxygen adj1 therap*) or (oxygen adj1 treatment*)).tw.)	24303

41	positive end expiratory pressure ventilation/ or ((continuous adj1 positive adj1 airway adj1 pressure) or (positive adj1 pressure adj1 respiration) or (bubble adj1 CPAP)).tw.	22005

42	exp physiotherapy/	107415

43	(physiotherap* or physical therap*).tw.	96671

44	((nasal* or nose or naso) adj3 suction*).tw.	183

45	suction/ or (deep adj1 suction*).tw.	12931

46	sodium chloride/na [Intranasal Drug Administration]	177

47	((saline adj1 drop*) or (nasal adj1 saline)).tw.	667

48	enteric feeding/ or exp parenteral nutrition/ or exp digestive tract intubation/	92632

49	(((non adj1 oral) or oral) and (feed* or hydration or fluid* or solution* or therap*)).tw.	337435

50	exp fluid therapy/ or oral rehydration solution/	112397

51	exp bacterial infection/	955606

52	exp otitis media/	38079

53	exp meningitis/	114158

54	exp *antiinfective agent/dt or (antibiotic* or 3z or aruzilina or atizor or azadose or azasite or azatril or azenil or azibiot or azibiot-neo or azimin or azithral or azithromycin or azitrocin or azitromax or azitromicin or azitromicina or aziwok or azomyne or aztrin or azydrop or azyter or azythromycin or bazyt or cp-62933 or cp-62993 or cp62933 or cp62993 or forcin or inedol or infectoazit or isv-401 or isv401 or kromicin or macrozit or mezatrin or octavax or ordipha or ribotrex or sumamed or sunamed or tobyl or tromix or trozocina or ultreon or vinzam or xithrone or xz-450 or xz450 or zaret or zarom or zetamax or zeto or zibramax or zifin or zimericina or zistic or zithromax or zithrox or zitinn or zitrim or zitrobifan or zitrocin or zitromax or zmax).tw.	977691

55	exp sepsis/	334492

56	exp urinary tract infection/	139560

57	exp tracheitis/	4182

58	(serious adj1 bacterial adj1 infection*).tw.	1891

59	infection control/ or patient care/ or isolation.tw.	787000

60	coronavirus disease 2019/ or experimental coronavirus disease 2019/	363085

61	(2019-novel or 2019nCoV or 2019-nCoV or COVID-19 or COVID19 or COVID-2019 or COVID2019 or CONVID-19 or CONVID19 or CORVID-19 or CORVID19 or CoV2 or CoV-2 or HCoV* or Ncov* or Ncorona* or Ncorono* or NcovChina* or NcovChinese* or NcovHubei* or NcovWuhan* or SARS2 or SARS-2 or SARScoronavirus2 or SARScoronavirus-2 or SARScoronovirus2 or SARScoronovirus-2 or SARSCov19 or SARSCov-19 or SARS-CoV-2 or SARSCoV-2 or SARSCoV2 or WN-CoV or WNCoV or wuhan-virus).tw.	418425

62	((pneumonia* or outbreak* or respiratory-illness* or respiratory-disease* or respiratory-symptom* or seafood-market* or food-market* or wildlife) and (Wuhan or China or Chinese or Hubei or Huanan)).tw.	23979

63	((new or novel or nouveau or ("length of stay" or hospital admission or hospital discharge or (criteria adj4 discharge)) or "2019" or Wuhan or Hubei or Huanan or China or Chinese) adj3 (coronavirus* or corona virus* or betacoronavirus* or CoV or HCoV)).tw.	85970

64	long COVID/ or (longCOVID* or postCOVID* or postcoronavirus* or postSARS*).tw.	4909

65	(coronavirinae/ or betacoronavirus/ or coronavirus infection/) and (epidemic/ or pandemic/)	9531

66	((coronavirus* or corona-virus* or betacoronavirus*) adj3 (pandemic* or epidemic* or outbreak* or crisis)).tw.	17154

67	severe-acute-respiratory-syndrome-coronavirus-2.hw.	97668

68	coronavirus-disease-2019.hw.	363661

69	exp Monoclonal antibody/ or (monoclonal-antibod* or clonal-antibod* or hybridoma-antibod* or nirsevimab or medi-8897 or medi8897 or sp-0232 or sp-232 or sp0232 or sp232 or Motavizumab or medi-524 or medi524 or numax or Palivizumab or abbosynagis or medi-493 or medi493 or synagis or synagys).tw.	833604

70	or/13-69	14210720

71	5 and 70	69784

72	pregnant woman/ or exp pregnancy/ or prenatal care/ or (parturition or ante-natal or antenatal* or pre-natal* or prenatal* or pregnan*).tw.	1159109

73	exp immunization/ or (immunis* or immuniz* or vaccin*).tw.	656913

74	(Respiratory-syncytial-virus-vaccine* or RSV-vaccine* or Arexvy).tw.	1061

75	(72 and 73 and 5) or (72 and 74)	512

76	(73 and 5) or 74	8119

77	12 or 71 or 76	71881

78	(newborn* or new-born* or baby or babies or neonat* or neo-nat* or infan* or toddler* or aged-1 or aged-one or one-year-old or 1-year-old or under-two or under-2 or younger-than-two or younger-than-2 or below-two or below-2 or under-24-months or younger-than-24-months or below-24-months or aged-1-to-23-months or aged-one-to-twenty-three-months).tw,kf,hw,dq.	1649049

79	bronchiolitis obliterans/ or (bronchiolitis adj1 obliterans).af.	10869

80	(77 and 78) not 79	20025

81	75 not 79	503

82	80 or 81	20142

83	limit 82 to (english language and embase and yr="2000 -Current")	11881

**Cochrane Library**

Original search date: 21/06/23

#1	MeSH descriptor: [Bronchiolitis] explode all trees

#2	bronchiolit* or wheez* or (Respiratory Syncytial Virus*) or rsv

#3	MeSH descriptor: [Respiratory Syncytial Viruses] explode all trees

#4	MeSH descriptor: [Respiratory Syncytial Virus Infections] explode all trees

#5	MeSH descriptor: [Natural History] explode all trees

#6	MeSH descriptor: [Epidemiology] explode all trees

#7	MeSH descriptor: [Severity of Illness Index] explode all trees

#8	MeSH descriptor: [Diagnosis, Differential] explode all trees

#9	MeSH descriptor: [Physical Examination] explode all trees

#10	MeSH descriptor: [Respiratory Tract Infections] explode all trees

#11	MeSH descriptor: [Risk Factors] explode all trees

#12	MeSH descriptor: [Length of Stay] explode all trees

#13	MeSH descriptor: [Patient Admission] explode all trees

#14	MeSH descriptor: [Intensive Care Units] explode all trees

#15	MeSH descriptor: [Morbidity] explode all trees

#16	MeSH descriptor: [Mortality] explode all trees

#17	MeSH descriptor: [Diagnostic Imaging] explode all trees

#18	MeSH descriptor: [Hematologic Tests] explode all trees

#19	MeSH descriptor: [Urinalysis] explode all trees

#20	MeSH descriptor: [Nasal Lavage Fluid] explode all trees

#21	MeSH descriptor: [Emergency Service, Hospital] explode all trees

#22	MeSH descriptor: [Albuterol] explode all trees

#23	MeSH descriptor: [Epinephrine] explode all trees

#24	MeSH descriptor: [Steroids] explode all trees

#25	MeSH descriptor: [Bronchodilator Agents] explode all trees

#26	MeSH descriptor: [Cholinergic Antagonists] explode all trees

#27	MeSH descriptor: [Anti-Inflammatory Agents] explode all trees

#28	MeSH descriptor: [Adrenal Cortex Hormones] explode all trees

#29	MeSH descriptor: [Leukotriene Antagonists] explode all trees

#30	MeSH descriptor: [Saline Solution, Hypertonic] explode all trees

#31	MeSH descriptor: [Aerosols] explode all trees

#32	MeSH descriptor: [Nebulizers and Vaporizers] explode all trees

#33	MeSH descriptor: [Sodium Chloride] explode all trees

#34	(#31 or #32) and #33

#35	MeSH descriptor: [Oxygen Inhalation Therapy] explode all trees

#36	MeSH descriptor: [Oxygen] explode all trees and with qualifier(s): [administration & dosage - AD, standards - ST]

#37	MeSH descriptor: [Oximetry] explode all trees

#38	MeSH descriptor: [Reproducibility of Results] explode all trees

#39	#37 and #38

#40	MeSH descriptor: [Physical Therapy Modalities] explode all trees

#41	MeSH descriptor: [Physical Therapy Specialty] explode all trees

#42	MeSH descriptor: [Suction] explode all trees

#43	MeSH descriptor: [Fluid Therapy] explode all trees

#44	MeSH descriptor: [Infusions, Intravenous] explode all trees

#45	MeSH descriptor: [Administration, Oral] explode all trees

#46	#43 and (#44 or #45)

#47	MeSH descriptor: [Administration, Intranasal] explode all trees

#48	saline (Word variations have been searched)

#49	#48 and #47

#50	MeSH descriptor: [Rehydration Solutions] explode all trees

#51	MeSH descriptor: [Bacterial Infections] explode all trees

#52	MeSH descriptor: [Otitis Media] explode all trees

#53	MeSH descriptor: [Meningitis] explode all trees

#54	MeSH descriptor: [Anti-Bacterial Agents] explode all trees and with qualifier(s): [therapeutic use - TU]

#55	MeSH descriptor: [Sepsis] explode all trees

#56	MeSH descriptor: [Urinary Tract Infections] explode all trees

#57	MeSH descriptor: [Tracheitis] explode all trees

#58	MeSH descriptor: [Radiography, Thoracic] explode all trees

#59	MeSH descriptor: [Asthma] explode all trees

#60	MeSH descriptor: [Hypersensitivity, Immediate] explode all trees

#61	MeSH descriptor: [Receptors, Adrenergic, beta-2] explode all trees

#62	MeSH descriptor: [Continuous Positive Airway Pressure] explode all trees

#63	MeSH descriptor: [Enteral Nutrition] explode all trees

#64	MeSH descriptor: [Parenteral Nutrition] explode all trees

#65	MeSH descriptor: [Intubation, Gastrointestinal] explode all trees

#66	MeSH descriptor: [Infection Control] explode all trees

#67	MeSH descriptor: [Primary Prevention] explode all trees

#68	MeSH descriptor: [Patient Isolation] explode all trees

#69	(History or Epidemiolog* or "severity of illness" or “disease severity” or scoring system* or diagnosis or physical exam* or auscultation or "blood pressure" or palpation* or percussion or pulse or vital sign* or Respiratory Tract Infection* or risk factor* or "length of stay" or admission or discharge or morbidit* or prevalence or mortalit* or "Diagnostic Imaging" or ((chest or thorac*) and (x-ray* or xray* or “x ray” or radiograph*)) or ((Hematolog* or Haematolog* or blood or virolog* or urine) and (Test or tests or exam* or investigation*)) or Urinalys* or “Nasal Lavage” or ((nose or nasal) and (mucosa or mucus)) or emergency department* or asthma* or atopy or atopic or hypersensitiv*) (Word variations have been searched)

#70	salbutamol or albuterol or ventolin or levalbuterol or adrenalin* or epinephrin* or beta2 adrenergic* or beta2 agonist* or ics or corticosteroid* or cortico steroid*OR cortico-steroid* or glucocorticoid* or gluco corticoid* or gluco-corticoid* or montelukast or Bronchodilat* or steroid or steroids or Cholinergic Antagonist* or cholinergic receptor* or Anti Inflammatory Agent* or Adrenal Cortex Hormone* or Leukotriene Antagonist* or Leukotriene receptor* or "Hypertonic Saline" or (("Sodium Chloride" or saline) and (nebuliz* or nebulis* or vaporiz* or vaporis* or aerosol* or intranasal or "intra nasal" or intra-nasal or nasal)) (Word variations have been searched)

#71	(Oxygen or ((Oximetry or oximeter*) and ("reproducibility of results" or reliability or validity or function* or technical specification* or percutaneous measurement* or blood gas analys*)) or CPAP or “continuous positive airway pressure” or “positive pressure respiration” or “positive end respiratory pressure”) (Word variations have been searched)

#72	Physical Therap* or physiotherap* or ((nasal* or nose or naso) and (suction* or toilet or irrigation)) or suction* or saline drop* or "nasal saline" or "intranasal saline" (Word variations have been searched)

#73	(Fluid Therap* or Intravenous infusion* or “non oral” or Rehydrat* or “enteric feeding” or “parenteral nutrition” or “parenteral feeding” or “enteral nutrition” or oral* administ* or bacterial infection* or "otitis media" or Meningitis or antibacterial agent* or anti bacterial agent* or antimicrobial agent* or anti microbial agent* or antibiotic* or 3z or aruzilina or atizor or azadose or azasite or azatril or azenil or azibiot or azibiot-neo or azimin or azithral or azithromycin or azitrocin or azitromax or azitromicin or azitromicina or aziwok or azomyne or aztrin or azydrop or azyter or azythromycin or bazyt or cp-62933 or cp-62993 or cp62933 or cp62993 or forcin or inedol or infectoazit or isv-401 or isv401 or kromicin or macrozit or mezatrin or octavax or ordipha or ribotrex or sumamed or sunamed or tobyl or tromix or trozocina or ultreon or vinzam or xithrone or xz-450 or xz450 or zaret or zarom or zetamax or zeto or zibramax or zifin or zimericina or zistic or zithromax or zithrox or zitinn or zitrim or zitrobifan or zitrocin or zitromax or zmax or Sepsis or septic or Urinary Tract Infection* or tracheitis or serious bacterial infection* or “infection control” or “primary prevention” or isolation or “patient care”) (Word variations have been searched)

#74	“intensive care” or ICU or "respiratory care" or NICU or PICU

#75	MeSH descriptor: [SARS-CoV-2] this term only

#76	MeSH descriptor: [COVID-19] this term only

#77	((corona* or corono*) NEAR (virus* or viral* or virinae*)) (Word variations have been searched)

#78	(coronavirus* or coronovirus* or coronavirinae* or CoV or CoV2 or CoV-2 or HCoV*) (Word variations have been searched)

#79	(“2019 nCoV” or 2019nCoV or nCoV2019 or nCoV-2019 or COVID-19 or COVID19 or CORVID-19 or CORVID19 or WNCoV or HCoV-19 or HCoV19 or (2019 NEXT novel*) or Ncov or SARSCoV-2 or SARSCoV2 or SARSCov19 or SARSCov-19 or Ncov or Ncorona* or Ncorono* or NcovWuhan* or NcovHubei* or NcovChina* or NcovChinese* or SARS2 or SARS-2 or SARScoronavirus2 or SARScoronavirus-2 or SARScoronovirus2 or SARScoronovirus-2)

#80	(respiratory* NEAR/2 (symptom* or disease* or illness* or condition*) NEAR/10 (Wuhan* or Hubei* or China* or Chinese* or Huanan*))

#81	((seafood-market* or food-market* or pneumonia*) NEAR/10 (Wuhan* or Hubei* or China* or Chinese* or Huanan*))

#82	((outbreak* or wildlife* or pandemic* or epidemic*) NEAR (Wuhan* or Hubei or China* or Chinese* or Huanan*))

#83	(COVID-2019 or COVID2019)

#84	MeSH descriptor: [Post-Acute COVID-19 Syndrome] this term only

#85	(longCOVID* or postCOVID* or postcoronavirus* or postSARS*)

#86	MeSH descriptor: [Antibodies, Monoclonal] explode all trees

#87	(monoclonal-antibod* or clonal-antibod* or hybridoma-antibod* or nirsevimab or medi-8897 or medi8897 or sp-0232 or sp-232 or sp0232 or sp232 or Motavizumab or medi-524 or medi524 or numax or Palivizumab or abbosynagis or medi-493 or medi493 or synagis or synagys)

#88	#5 or #6 or #7 or #8 or #9 or #10 or #11 or #12 or #13 or #14 or #15 or #16 or #17 or #18 or #19 or #20 or #21 or #22 or #23 or #24 or #25 or #26 or #27 or #28 or #29 or #30 or #34 or #35 or #36 or #39 or #40 or #41 or #42 or #46 or #49 or #50 or #51 or #52 or #53 or #54 or #55 or #56 or #57 or #58 or #59 or #60 or #61 or #62 or #63 or #64 or #65 or #66 or #67 or #68 or #69 or #70 or #71 or #72 or #73 or #74 or #75 or #76 or #77 or #78 or #79 or #80 or #81 or #82 or #83 or #84 or #85 or #86 or #87

#89	(#1 or #2 or #3 or #4) and #88

#90	MeSH descriptor: [Pregnant Women] explode all trees

#91	MeSH descriptor: [Pregnancy] explode all trees

#92	MeSH descriptor: [Prenatal Care] this term only

#93	(parturition or ante-natal or antenatal* or pre-natal* or prenatal* or pregnan*)

#94	#90 OR #91 or #92 or #93

#95	MeSH descriptor: [Immunization] explode all trees

#96	(immunis* or immuniz* or vaccin*)

#97	#95 OR #96

#98	Respiratory-syncytial-virus-vaccine* or RSV-vaccine* or Arexvy

#99	(#94 and #97 and (#1 or #2 or #3 or #4)) or (#94 and #98)

#100	(#97 and (#1 or #2 or #3 or #4)) or #98

#101	#89 or #100

#102	(newborn* or new born* or baby or babies or neonat* or neo nat* or infan* or toddler* or “aged 1” or “aged one” or “one year old” or “1 year old” or “under two” or “under 2” or “younger than two” or “younger than 2” or “below two” or “below 2” or “under 24 months” or “younger than 24 months” or “below 24 months” or “aged 1 to 23 months” or “aged one to twenty three months”)

#103	MeSH descriptor: [Bronchiolitis Obliterans] explode all trees

#104	("bronchiolitis obliterans")

#105	(#101 and #102) not (#103 or #104)

#106	#99 not (#103 or #104)

#107	#105 or #106 with Cochrane Library publication date from Jan 2000 to present

**Results = 2164**

**CINAHL**

Original search date 21/06/23

S20	S18 AND S19

Limiters - English; Published Date: 20000101-; Peer Reviewed	(56)

S19	newborn* or new-born* or baby or babies or neonat* or neo-nat* or infan* or toddler* or aged-1 or aged-one or one-year-old or 1-year-old or under-two or under-2 or younger-than-two or younger-than-2 or below-two or below-2 or under-24-months or younger-than-24-months or below-24-months or aged-1-to-23-months or aged-one-to-twenty-three-months	(553,820)

S18	S9 AND S17	(114)

S17	S10 OR S11 OR S12 OR S13 OR S14 OR S15 OR S16	(202,865)

S16	(saline W1 drop*) or (nasal W1 saline)	(104)

S15	(MH "Administration, Intranasal") and saline	(145)

S14	physiotherap* or "physical therap*"	(87,362)

S13	(MH "Physical Therapy+")	(159,654)

S12	(MH "Suctioning, Nasopharyngeal") OR (MH "Suction") OR (deep W1 suction*) OR ((nasal* or nose or naso) W3 suction*)	(2,755)

S11	(MH "Rehabilitation, Pulmonary")

Limiters - Published Date: 20000101-20011231	(106)

S10	(MH "Chest Physiotherapy (Saba CCC)") OR (MH "Chest Physiotherapy (Iowa NIC)") OR (MH "Chest Physical Therapy+")	(870)

S9	(s8 not s5) or s6 or s7	(8,657)

S8	S1 OR S2 OR S3 OR S4	(10,610)

S7	(S1 OR S2 OR S3 OR S4) and (MH "Professional Practice, Evidence-Based+")	(108)

S6	S1 OR S2 OR S3 OR S4

Limiters - Publication Type: Clinical Trial, Critical Path, Meta Analysis, Practice Guidelines, Randomized Controlled Trial, Review, Systematic Review	(1,501)

S5	S1 OR S2 OR S3 OR S4

Limiters - Publication Type: Case Study, Commentary, editorial, Letter	(2,006)

S4	bronchiolit* or wheez* or "respiratory syncytial virus*" or rsv	(10,438)

S3	(MH "Respiratory Syncytial Virus Infections") OR (MH "Respiratory Syncytial Viruses")	(2,772)

S2	(MH "Bronchial Diseases")

Limiters - Published Date: 20000101-20001231	(40)

S1	(MH "Bronchiolitis+")	(2,540)

**PubMed**

Original search date 21/6/23

#1	bronchiolitis OR bronchiolitic OR respiratory-syncytial-virus* OR wheez* OR rsv

#2	History OR Epidemiolog* OR "severity of illness" OR "disease severity" OR "scoring system" OR diagnosis OR physical-exam* OR auscultation OR "blood pressure" OR palpation* OR percussion OR pulse OR vital-sign* OR Respiratory-Tract-Infection* OR risk-factor* OR "length of stay" OR admission OR discharge OR "intensive care" OR NICU OR ICU OR PICU OR "respiratory care" OR morbidit* OR prevalence OR mortalit* OR "Diagnostic Imaging" OR ((chest OR thorax OR thoracic) AND (x-ray* OR xray* OR radiograph*)) OR ((Hematolog* OR Haematolog* OR blood OR virolog* OR urine) AND (Test OR tests OR exam* OR investigation*)) OR Urinalys* OR "Nasal Lavage" OR ((nose OR nasal) AND (mucosa OR mucus)) OR emergency-department* OR asthma* OR atopy OR atopic OR hypersensitiv* OR salbutamol OR albuterol OR ventolin OR levalbuterol OR adrenalin* OR epinephrin* OR beta2-adrenergic* OR beta2-agonist* OR ics OR corticosteroid* OR cortico-steroid* OR glucocorticoid* OR gluco corticoid* OR montelukast OR Bronchodilat* OR steroid OR steroids OR Cholinergic-Antagonist* OR cholinergic-receptor* OR Anti-Inflammatory-Agent* OR Adrenal-Cortex-Hormone* OR Leukotriene-Antagonist* OR Leukotriene-receptor* OR "Hypertonic Saline" OR (("Sodium Chloride" OR saline) AND (nebuliz* OR nebulis* OR vaporiz* OR vaporis* OR aerosol* OR intranasal OR intra-nasal OR nasal)) OR Oxygen OR ((Oximetry OR oximeter*) AND ("reproducibility of results" OR reliability OR validity OR function* OR technical-specification* OR percutaneous-measurement* OR blood-gas-analys*)) OR CPAP OR "continuous positive airway pressure" OR "positive pressure respiration" OR "positive end respiratory pressure" OR Physical-Therap* OR physiotherap* OR ((nasal* OR nose OR naso) AND suction*) OR suction* OR saline-drop* OR "nasal saline" OR "nasal toilet" OR "nasal irrigation" OR Fluid-Therap* OR Intravenous-infusion* OR "non oral" OR Rehydrat* OR "enteric feeding" OR "parenteral nutrition" OR "parenteral feeding" OR "enteral nutrition" OR oral* AND administ* OR bacterial-infection* OR "otitis media" OR Meningitis OR antibacterial-agent* OR anti-bacterial-agent* OR antimicrobial-agent* OR anti-microbial-agent* OR antibiotic* OR 3z OR aruzilina OR atizor OR azadose OR azasite OR azatril OR azenil OR azibiot OR azibiot-neo OR azimin OR azithral OR azithromycin OR azitrocin OR azitromax OR azitromicin OR azitromicina OR aziwok OR azomyne OR aztrin OR azydrop OR azyter OR azythromycin OR bazyt OR cp-62933 OR cp-62993 OR cp62933 OR cp62993 OR forcin OR inedol OR infectoazit OR isv-401 OR isv401 OR kromicin OR macrozit OR mezatrin OR octavax OR ordipha OR ribotrex OR sumamed OR sunamed OR tobyl OR tromix OR trozocina OR ultreon OR vinzam OR xithrone OR xz-450 OR xz450 OR zaret OR zarom OR zetamax OR zeto OR zibramax OR zifin OR zimericina OR zistic OR zithromax OR zithrox OR zitinn OR zitrim OR zitrobifan OR zitrocin OR zitromax OR zmax OR Sepsis OR septic OR Urinary-Tract-Infection* OR tracheitis OR serious-bacterial-infection* OR "infection control" OR "primary prevention" OR isolation OR "patient care" OR monoclonal-antibod* OR clonal-antibod* OR hybridoma-antibod* OR nirsevimab OR medi-8897 OR medi8897 OR sp-0232 OR sp-232 OR sp0232 OR sp232 OR Motavizumab OR medi-524 OR medi524 OR numax OR Palivizumab OR abbosynagis OR medi-493 OR medi493 OR synagis OR synagys OR (2019-novel OR 2019nCoV OR 2019-nCoV OR COVID-19 OR COVID19 OR COVID-2019 OR COVID2019 OR CONVID-19 OR CONVID19 OR CORVID-19 OR CORVID19 OR CoV2 OR CoV-2 OR HCoV* OR Ncov* OR Ncorona* OR Ncorono* OR NcovChina* OR NcovChinese* OR NcovHubei* OR NcovWuhan* OR SARS2 OR SARS-2 OR SARScoronavirus2 OR SARScoronavirus-2 OR SARScoronovirus2 OR SARScoronovirus-2 OR SARSCov19 OR SARSCov-19 OR SARS-CoV-2 OR SARSCoV-2 OR SARSCoV2 OR WN-CoV OR WNCoV OR wuhan-virus) OR ((pneumonia* OR outbreak* OR respiratory-illness* OR respiratory-disease* OR respiratory-symptom* OR seafood-market* OR food-market* OR wildlife) AND (Wuhan OR China OR Chinese OR Hubei OR Huanan)) OR ((new OR novel OR nouveau OR 19 OR 2019 OR Wuhan OR Hubei OR Huanan OR China OR Chinese) AND (coronavirus* OR corona virus* OR betacoronavirus* OR CoV OR HCoV)) OR (longCOVID* OR postCOVID* OR postcoronavirus* OR postSARS*) OR ((coronavirus* OR corona-virus* OR betacoronavirus*) AND (pandemic* OR epidemic* OR outbreak* OR crisis))

#3	newborn* OR new-born* OR baby OR babies OR neonat* OR neo-nat* OR infan* OR toddler* OR aged-1 OR aged-one OR one-year-old OR 1-year-old OR under-two OR under-2 OR younger-than-two OR younger-than-2 OR below-two OR below-2 OR under-24-months OR younger-than-24-months OR below-24-months OR aged-1-to-23-months OR aged-one-to-twenty-three-months

#4	NOTNLM

#5	"Bronchiolitis Obliterans"

#6	(#1 AND #2 AND #3 AND #4) NOT #5

Limit 2000 onwards; AND English	2168

[Search name: Cate Wilson 300623 Bronchiolitis AND VOI AND Age AND NLM]

#7	#1 AND #2 AND #3) NOT #5

Limit 2013 onwards; AND English	3576

[Search name: Cate Wilson 300623 Bronchiolitis AND VOI AND Age]

#8	((bronchiolitis OR bronchiolitic OR respiratory-syncytial-virus* OR wheez* OR rsv) AND (parturition OR ante-natal OR antenatal* OR pre-natal* OR prenatal* OR pregnan*) AND (immunis* OR immuniz* OR vaccin*)) OR ((parturition OR ante-natal OR antenatal* OR pre-natal* OR prenatal* OR pregnan*) AND (Respiratory-syncytial-virus-vaccine* OR RSV-vaccine* OR Arexvy))

#9	(#8 AND #4) NOT #5

Limit 2000 onwards; AND English	160

[Search name: Cate Wilson 300623 Bronchiolitis AND Maternal immunization AND NLM]

#10	#8 NOT #5

Limit 2013 onwards; AND English	271

[Search name: Cate Wilson 300623 Bronchiolitis AND Maternal immunization]

#11	((bronchiolitis OR bronchiolitic OR respiratory-syncytial-virus* OR wheez* OR rsv) AND (immunis* OR immuniz* OR vaccin*)) OR (Respiratory-syncytial-virus-vaccine* OR RSV-vaccine* OR Arexvy)

#12	(#11 AND #3 AND #4) NOT #5

Limit 2000 onwards; AND English	1087

[Search name: Cate Wilson 300623 Bronchiolitis AND Infant immunization AND Age AND NLM]

#13	(#11 AND #3) NOT #5

Limit 2013 onwards; AND English	1725

[Search name: Cate Wilson 300623 Bronchiolitis AND Infant immunization AND Age]

#14	#6 OR #7 OR #9 OR #10 OR #12 OR #13	4384

## **Supplementary Appendix A3. GRADE certainty of evidence tables**

**Author:** PREDICT research network

**Question:** For infants presenting to hospital or hospitalised with bronchiolitis, what criteria should be used for safe discharge?

**Settings:** Hospital

**Bibliography:** Garcia-Mauriño 2018; Gilbert 2023.

| Quality assessment | | | | | | | No. of patients | Key findings | Quality | Importance |
| --- | --- | --- | --- | --- | --- | --- | --- | --- | --- | --- |
| No. of studies | Design | Risk of bias | Inconsistency | Indirectness | Imprecision | Other considerations |  |  |  |  |
| **Length of stay** (Median length of hospital stay in days) | | | | | | | | | | |
| 1^1^ | Retrospective observational | Very serious^2^ | NA | Serious^3^ | Not serious | None | n=1,118 discharged from ID unit  n=695 discharged from non-ID units | ID unit (with protocol) vs. non-ID units (without protocol):  Including PICU stay: 2.0 days (IQR 1.4 to 3.8) vs. 2.8 days (IQR 1.7 to 4.9), ***p*<.001**.  ^4^Adj RR= 1.35, CI= 1.27 to 1.44, ***p*<.001**.  Excluding PICU stay: 1.7 days (IQR 1.1 to 2.5) vs. 2 days (1.2 to 3.3), ***p*<.001**. | ⊕⊝⊝⊝  Very low | Critical |
| 1^5^ | Retrospective cohort | Very serious^6^ | NA | Not serious | Not serious | None | n=462 received revised protocol  n=422 received prior protocol | Prior protocol vs. revised protocol:  Median 2.11 days (IQR 1.54 to 2.97) vs. 1.83 days (IQR 1.17 to 2.71), ***p*<.001**.  Median difference -6.7 hours. | ⊕⊕⊝⊝  Low | Critical |
| **Readmission rate** (Number of patients readmitted to hospital within two weeks of discharge) | | | | | | | | | | |
| 1^1^ | Retrospective observational | Very serious^2^ | NA | Serious^3^ | Serious^7^ | None | n=1,118 discharged from ID unit  n=695 discharged from non-ID units | ID unit (with protocol) vs. non-ID units (without protocol):  33/1118 (2.9%) vs. 18/695 (2.6%), *p*=.77.  ^8^Adj OR= 1.08= CI: 0.57 to 1.97, *p*=.86.  2 more per 1,000 (from 11 fewer to 24 more). | ⊕⊝⊝⊝  Very low | Critical |
| **Readmission rate** (Number of patients readmitted to hospital within five days of discharge with a bronchiolitis diagnosis and respiratory support requirement) | | | | | | | | | | |
| 1^5^ | Retrospective cohort | Very serious^6^ | NA | Serious^9^ | Not serious | None | n=462 received revised protocol  n=422 received prior protocol | Prior protocol vs. revised protocol:  3 of 462 (0.6%) vs. 6 of 422 (1.4%), *p*=.317. | ⊕⊝⊝⊝  Very low | Critical |
| CI= confidence interval; ID= infectious disease; IQR= interquartile range; LOS= length of stay; NA= not applicable; OR= odds ratio; PICU= paediatric intensive care unit; RR= risk ratio.  ^1^ Garcia-Mauriño 2018  ^2^ Very serious risk of bias. Downgraded due to critical concerns about confounding and some concerns about selection of the reported result, that were sufficient to substantially lower confidence in the estimate of effect.  ^3^ Serious indirectness. Downgraded due to indirectness in the setting. Single tertiary paediatric hospital study from the USA comparing “ID” unit to “non-ID” units. Management of bronchiolitis in Australiasia is within different healthcare settings and predominantly in general paediatric wards in non-tertiary hospitals.  ^4^ Value for all infants (with and without comorbidity, with and without PICU admission), who were exposed versus not exposed to the discharge protocol.  ^5^ Gilbert 2023.  ^6^ Very serious risk of bias. Downgraded due to serious concerns about confounding, and some concerns about selection of the reported result.  ^7^ Serious imprecision. Downgraded due to wide CIs.  ^8^ Value for all infants (with and without comorbidity), who were exposed versus not exposed to the discharge protocol.  ^9^ Serious indirectness. Downgraded due to indirectness in the outcome measure, as only readmissions for bronchiolitis with a respiratory support requirement were counted.  NB*.* As only observational evidence was sought for this question, a high quality rating was possible with observational studies. | | | | | | | | | | |
